# Reports of the *Pestilential Disorders* of Andalusia, Which Appeared at Cadiz in the Years 1800, 1804, 1810, and 1813; with a Detailed Account of the Fatal Epidemic as It Prevailed at Gibraltar during the Autumn of 1804, &c. &c. &c.

**Published:** 1816-01

**Authors:** 


### PART II.

### COMPREHENSIVE ANALYTICAL REVIEW OF MEDICAL LITERATURE.

"Tros, tyriusve nobis nullo discrimine agetur."

*Reports of the* PESTILENTIAL DISORDERS *of Andalusia, which appeared at Cadiz in the Years* 1800, 1804, 1810, *and* 1813; *with a detailed Account of the fatal Epidemic as it prevailed at Gibraltar during the Autumn of* 1804, *&c. &c. &c.* By SIR JAMES FELLOWES, M. D. Physician to the Forces, &c. &c. 8vo. pp. 484.

WHEN the livid hand of pestilence presses heavily on any portion of the human race, however remotely situated, is there any one so immersed in the toils, or absorbed in the profits of his profession, as not to lend an ear of sympathy, or at least of curiosity, to the disastrous tale? Sunt lachrymæ rerum, et mentem *mortalia* tangunt!

The reading and the thinking part of the medical community in England, have long complained of the mysterious silence which our army practitioners maintained, till very lately, respecting the fevers which ravaged the Southern Parts of Spain since the beginning of the present century; but the blank is now pretty well filled up, though the discrepancies of opinion are as great as ever! The volume of Mr. Pym, which we lately reviewed in the concluding part of our first decade, though embracing a great variety of subjects, and particularly those of the present work, was chiefly *disputative,* and did not, by any means present either so instructive or interesting a picture of the great events described, as the volume now under review. Sir James Fellowes, however, is entirely on the same side of the question as Mr. Pym, and therefore we shall endeavour, as much as possible, to avoid a *repetition* of those arguments and opinions in favour of contagion and of a disease *sui generis,* which were so amply displayed and discussed in the review of Mr. Pym. We hope, nevertheless, to present our readers with such a condensed view of the medical topography and fevers of Andalusia, as cannot fail to arrest their attention, excite their sympathy, and increase their store of knowledge, during its perusal.

The present subject is comprised in four Reports; the fifth being occupied with observations on the Walcheren fever. We shall follow Sir James Fellowes's divisions seriatim.

### Report 1.—Part I. Medical Topography of Cadiz.

The area on which this city stands is about a square mile, raised above the level of the *nearly* surrounding ocean, from nine to forty-seven feet. The superficies of the soil is pure sand, except where crusts of the subjacent rock occasionally appear. The population in 1813, when the last epidemic prevailed, was computed at 70,000 souls. Since 1800, the dead bodies have been buried in a cemetery, a mile from the town. The situation of Cadiz is singularly striking. On making it from the westward, the city seems to rise majestically from the ocean, the nearest high land being the Sierras of Xeres and Ronda, distant fifteen leagues. Cadiz, which was anciently supplied with fresh water by the famous aqueduct from Tempul, has now nothing but wells and *morias* of brackish water within its walls. Every house, however, has its *algibe* or cistern, in which water is collected from the flat terraces during rains; in defect of which, water is brought from Puerto de St. Maria in boats. Cadiz was anciently celebrated for the salubrity of its air, and fertility of its soil, but at present there is not much occasion to boast of either!

The most crowded and ill-ventilated part of Cadiz is the district of St. Maria, where the posadas, taverns, and lodging houses for the lowest classes of inhabitants are situated, and where epidemic diseases first break out in general.

Each house consists of three or four stories, terminating in a flat brick roof *(azotea)* or terrace, on which are erected towers of stone or painted wood, with turrets and ornaments in the Moorish fashion, that produce a most picturesque effect when viewed from the bay. The buildings are constructed with a multiplicity of doors and windows, that admit of a very free circulation of air, while the elevation of the peninsula above the level of the sea, offers natural facilities for public drains to cross the town in all directions, rendering it one of the cleanest and neatest cities of Europe.

During summer, sea and land breezes prevail, the former from the N. W. quarter, the latter from the North East. These, in winter, are changed into violent South West gales, accompanied often by heavy storms of rain. In summer, the *Levanters* alternate with the sea and land breezes, to the great annoyance of the inhabitants. This wind, which is so hot and dry as to scorch the tender plants and ripening corn, covers the neighbouring mountains with a heavy dark mist. At this time the circulating system in the human frame becomes wonderfully deranged. " The fibres are irritated ; the quality of the bile itself is altered, and the most pacific, quiet temper is rendered irritable." This wind comes in a southerly direction over the scorching plains of Africa. Cholera morbus ; bilious diarrhœas ; violent pain and giddiness of the head, and even fatal apoplexies are the consequences of these levanters. The Andalusian fever has always been much influenced, if not occasioned by this wind. The atmosphere of Cadiz is very moist, every wind that blows coming over the sea, and being charged with humidity. In respect to diet, fish is the favourite food of the natives; in defect of which, soup, and the national dish commonly called *olla*, is the usual diet. It may be remarked, however, that the opulent indulge in gross, while the poor are forced on innutritious and unwholesome aliment, both classes exhibiting sickly constitutions, while there is no where to be seen the dark, but manly complexion of the Spanish nation. Upon the whole, although the climate cannot be called insalubrious, yet the circumscribed space into which so vast a population is crowded, together with the influence of sedentary habits and fluctuating passions, must operate in predisposing to, and aggravating a number of diseases, and particularly fevers of an epidemic character.

The following is a list of comparative mortality during the first six years of the present century. 1801, 2,359; 1802, 2,809 ; 1803, 2,463; 1804, (the year of epidemic fever) 4,751 ; 1805, 2,723 ; 1806, 2,726.

### Part II. Origin and Progress of the Cadiz Fever in 1800.

The first great sickness that nearly depopulated Cadiz appeared in 1466. A similar event took place in 1507. The epidemic of 1582 is *said* to have ceased through the intercession of St. Roque! In 1649, the plague was introduced into Cadiz, and raged three years, carrying off more than 14,000 persons. It was not till the year 1730, that the disorder since known by the name of "El vomito negro," or black vomit, first made its appearance, and destroyed great numbers of the inhabitants. In the year following, it was equally dreadful, exhibiting spots of a livid, yellow, or dark colour, that covered the body, and were the certain forerunners of the black vomit. Don Josef Cervi, physician to Charles the Third declared, that it was *not* the plague; but Don F. Navarette affirmed, that this disease (el vomito negro) was introduced into Cadiz by a vessel from Spanish America, and that it spread to other parts of the Peninsula. Thus we see how early the discrepancies of opinion began, and how late they have continued!

In 1764, a similar disorder appeared in Cadiz, which was witnessed by our immortal countryman, Dr. James Lind. Salvaresa, a famous Spanish physician, has written a Latin, and Dr. Lind an English account of the disease; they both prove the disease to be one and the same with the late fatal epidemic, but Salvaresa says nothing of its being contagious; on the contrary, he attributes it to atmospherical causes and corrupted corn; while Lind so wavers in his opinion, that nothing decisive can be gleaned from him.

During a period of thirty-six years after the above fever, Cadiz remained healthy, notwithstanding its progressive increase of population. The last months of 1799, and the first five of 1800 were characterised by remarkably severe weather, so that at the end of May, there was scarcely any appearance of spring. All at once, however,

> "The heat of summer set in from the *beginning* of June, and by the month of August, the mercury was at 90° Fahrenheit's; while the *Levanter* tended to increase the distress which the intense heat of the weather generally occasioned." p. 33.

During the months of June and July, no material alteration took place in the state of the public health; but in the beginning of August, the scene began to change. A fever broke out in the district of St. Maria, which we have mentioned above, as the residence of poverty and theatre of filth. From this point it radiated in all directions; and in whatever house it appeared, all the family was ultimately attacked. A general consultation of the medical practitioners was now called, where violent discussions arose that led to nothing, but added to the universal confusion and dismay! The prevailing disorder was attributed to various causes, and was variously denominated, but the anticontagionists were evidently superior in number, for no precautions were taken against contagion. Sir James has brought forward a curious note of Aregula on this occasion. This great author asserts in triumph, that "two physicians, (Far and Navas) who most vehemently maintained that the prevailing disorder was not contagious, affirming that they had not lost a patient, were *the first who died of the fever in question.*" Aregula forgets that numbers had died before there was any discussion on the subject, yet with matchless effrontery sets down the two physicians abovementioned as the *first* victims of the disease! Oh, Sir James, this was an unfortunate note from your good friend Aregula.

About the middle of August, when the daily mortality amounted to twenty-five or thirty, all heads were laid together to find out the source of the evil, and they soon pitched upon a ship (the Dolphin) from Spanish America, where it seems some smugglers had received the infection and subsequently spread it through the town. This story is so extremely weak in itself, and badly put together, that we shall not attempt to trace it; but we earnestly recommend a careful consideration of the very unusual *atmospherical* phænomena which preceded this epidemic, before it be finally arranged in the class of imported contagions.

On the 23d of August, the image of our Saviour was carried out to appease the anger of the Deity, while the concourse and conflicting passions of the vast assemblages of people rapidly augmented the mortality to 157 per diem! By the middle of September, the diurnal mortality amounted to 200, and Cadiz presented the most melancholy scene of mourning and desolation! At this time the *disease* spread to domestic and other animals, and dogs and cats were seen dying with the black vomit. The very horses died! Will any man in his cool, unbiassed moments of reflection believe, that the *fever* of these animals was wafted across the Atlantic within the sides of the Dolphin ? *

The first check to the epidemic was the appearance of Lord Keith's hostile fleet, which, early in October, menaced a descent on the desolated city of Cadiz. We well remember the time. We saw the signal fly for the disembarkation. We saw the British bayonets bristle in the bay, ready to drink the blood of the wretched garrison and citizens. But the great hand of Nature interposed.— The conflicting elements burst forth in a hurricane that purged the city of " plague, pestilence, and famine," and dispersed, in a thousand directions, the invading fleet !

The neighbouring towns of Puerto Real, Port St. Mary's, Chulana, Rota, &c. being afflicted equally as much as Cadiz, the fever was, *of course,* traced to the emigrants from the latter city. Between the beginning of August and first week in November, 48,688 people were attacked by the epidemic in Cadiz, of which number 7,292 died. In Seville the mortality exceeded 22,000; in Xeres, 10,000. Thus ends the history of the Epidemic.

### Part III. Description of the Fever.

We think it necessary to apprise our readers, that none of the above circumstances are related by Sir James from *personal observation.* Our author saw no more of the disease than we did ; indeed, not so much, for *we* were very near its focus. Aregula is our author's sheet anchor on all occasions ; had it not been for whom, the volume before us would have cut but a meagre figure among modern octavo's.

The symptoms of this epidemic are divided into two classes—regular and irregular. The former gave no warning, but invaded the patient suddenly ; sometimes between four and twelve o'clock in the morning, but *generally* in the night. Chilliness ; violent pain across the forehead and temples ; in the loins, and lower extremities ; lassi-

---

* " The *air* from its *stagnant* state became so *vitiated,* that its *noxious qualities* affected even animals." p. 45. We would ask Dr. F. what these noxious qualities were ? Were they merely the consequence of *quiescence* in the atmosphere, or were they the consequence of pollution from *exhalations* mixed therein ? The *former* cannot be maintained, and the *latter* proves that *febrific* miasmata may exist *without marshes.*

Perhaps the contagionists may attempt to shew, that the " stagnant state of the air" was also imported from America in the Dolphin. If so, she must have had a *long passage !*

tude; yellow colour of the skin, especially about the third day. The countenance assumed a faded appearance. The cornea opaca of a red yellow. Restlessness; deliquum on standing up. The pulse was very irregular. To loss of appetite succeeded vomiting, but seldom diarrhœa; pain at the pit of the stomach; sweat and urine entirely bilious. A remission of these symptoms took place at the end of twenty or twenty-four hours, with an exacerbation the next day. Then a remission, or apparent apyrexia on the third, sometimes on the fourth, fifth, and seventh day, the latter very rare. During this time, the animal functions were seldom disturbed; but debility and anorexia continued long after the fever had disappeared. The irregular or anomalous signs of this disease were as follow: Sensation of cold or rigor; dull pain of the head and eyes, which appeared swollen, heavy, and extremely red. The heat of the body natural; tongue tremulous and dry, with a dark stripe in the middle. Insensibility; frequent efforts to vomit; weight and uneasiness about the region of the liver; pain and burning heat at the pit of the stomach. Change of colour to a leaden hue. Coldness of upper and lower extremities; continued or interrupted vomitings, first of a bilious, afterwards of an atrabilious matter; in other cases entirely black. The discharges from the bowels of the same colour, appearing like ground charcoal. Perpetual jactation; violent pain in the throat; difficult deglutition; deafness; red, black, or livid spots, especially on the parts pressed on. Yellowness; discharge of black blood from the mouth, nostrils, anus, eyes, ears, &c. were the precursors of death. The following were always *fatal* symptoms. Dark-red, or sublivid colour of the tongue. Darkness under the eyes; suppression of urine; irritation of the urethra, about the glans penis, forcing the patient to squeeze the penis.

Some patients had violent mad delirium, others continued in a comatose state. Many preserved a firmness of mind to the last moment of their lives. Several had tumors; three had carbuncles. A cutaneous eruption was common, and considered favourable. It spared those in advanced age, as also newly-born infants and young children. People of soft skins and mild dispositions escaped better than those of an opposite description: females experienced a greater immunity than males. Those about the age of puberty, of strong constitutions and dark complexions, experienced the most violent attacks; but to the pusillanimous, it was most fatal. Those born in hot climates had the advantage over those from cold countries. Early yellowness on the skin was an indifferent symptom; after the sixth day, it was favourable. Rigor, or sensation of *intense* cold, at the beginning was unfavourable, as was the total absence of sensation of cold. Regular and lasting shivering fits afforded the best presages. Severe pain at the pit of the stomach was a bad sign, especially if accompanied with dark-coloured vomitings. Few cooks escaped the disorder. The earlier the black vomit the greater the danger. "*Relapses were very frequent and fatal.*" Yet Aregula asserts, that the yellow fever of Andalusia only attacks persons once in their lives.

*Appearances on Dissection.*— Black or coffee-coloured matter in the stomach, duodenum, and colon. Gangrenous spots on the same viscera. Liver enlarged, and altered in appearance, approaching to the hue between yellow and black. Lungs speckled with black and gangrenous spots. "It was not uncommon to find some parts of the brain livid." Yellow colour of the skin, fat, and secretions very common. No other anatomical investigations were made.

Thus ends the Cadiz Epidemic of 1800. Nothing is there said respecting the treatment, but some general observations on this head will be introduced hereafter. We shall here, however, insert a passage from the *conclusion* of Sir James's Work, which does not exactly quadrate the opinions delivered above.

"As far as my experience goes, I should be induced to believe, that human contagion having acquired a concentrated virulence from a combination of *peculiar circumstances, joined to the epidemic tendency of the bilious remittent of the country,* gave rise to the pestilential disorder in Spain. It is possible, that persons coming from Vera Cruz or the Havanna, and carrying with them the seeds of *disease, admitted to be endemic in those places,* might, during their passage in a crowded ship, undergo such a change of constitution as to produce the disorder, with the *additional* property of generating it in others highly predisposed." p. 403.

Now, passing over some obscurity and confusion which run through the above passage, we appeal to our readers, whether it is not an acknowledgment of the truth of the doctrine which we have so often maintained, particularly in our reviews of Burnett and Pym. We also appeal to our readers, whether it is not almost a literal copy of the following passage.

"As far as my own observations and judgmeut could guide me, I have been led to conclude, that the *endemic* fevers are *not* contagious till a certain number of patients are confined together under *peculiar circumstances*, when the effluvia may render them so. If great numbers are attacked nearly at the same time, and confined in the sick-berth of a ship, it is possible, that a contagious atmosphere may be formed, which spreads a disease, wearing the livery of the prevailing endemic, but having a dangerous character superadded, namely, the power of reproducing itself in other subjects." —Johnson on Hot Climates, p. 108.

Whether the above passages be a *coincidence of opinion* or not, it is certain, that Sir James Fellowes is no longer to be ranked on the side of Dr. Chisholme or Mr. Pym, but as agreeing with the *neutral* party, of which we profess ourselves members. We must be allowed, however, to think, that a considerable change took place in our author's opinions *during* the construction of the volume before us; but *how or why* this change took place, is best known to the author himself.

Sir James next enters into a long detail of the topography of Gibraltar, and the history of the fever of 1804. The pages of the New Medical Journal have of late contained so much information on this subject, that we deem it unnecessary to *repeat* it here; for Sir James treads in the steps, and embraces the same opinions as Mr. Pym, in opposition to Dr. Burnett and Mr. Amiel. He is, however, much more minute than Mr. Pym, though we cannot say that he has at all *increased* our credence in contagion creed; nor have his arguments at all convinced us, that the disease was imported from Cadiz in the person of the *now* celebrated Santos. We shall here, however, copy a note from Sir James, relative to the topography of the spot from whence this fever radiated.

"The unhealthy circumstances to which Colonel Colville alludes, was the public drain or sewer running from the barracks; which, from being uncovered, and from the want of water to cleanse it, was, during the heats of summer, *extremely offensive*; it was, at *this time, particularly so*, the barrack necessaries having been emptied into it, and the *contents not having then run off*. The huts, in which so many sick inhabitants, and some of the married people were living, were built *adjoining the sewer*, and some *immediately over it*, with a *single boarded floor intervening*. The disorder prevailed *particularly in this spot*." 139.

It would hardly be believed, that the man who could write this passage, would gravely inform us, that "marsh miasmata" exist at Gibraltar! There is a disingenuous *quibble* always laid hold of by the contagionists respecting *vegeto-animal* effluvia. If they can prove that there is no *swamp* or *marsh*, such as we meet with in Zealand or Essex, they triumphantly exclaim—"here are no *marsh* miasmata, and consequently no endemic fever"! This is one instance of the importance of a *name*:—Vegeto-animal, or *febrific* effluvia, will as assuredly spring from the source described above by Sir James, as from the plains of Zealand or the sedgy shores of the Ganges. What madness of *party* is it then, to cling to the name of *marsh miasma* as an argument against endemic disease.

Those divisions of the work before us on the fever of Malaga, in 1803-4, are not very interesting. According to Aregula, (our author's sheet anchor on all occasions) the *infection* was brought to Malaga in four vessels; one from Smyrna, two from Marseilles, and one from Monte Video! Sir James traces it from a point in the *low dirty quarter*, or district of Perchel, which is often overflowed by the Guadalmedina.

"Even with the least torrent of the Guadalmedina, the streets are overflown; which, upon the waters retiring, are left full of mud and clay." 158.

Upon the symptoms of the fever we shall not dilate. They were by no means exactly similar to those of the fever at Cadiz and Gibraltar; though, if they all resulted from one specific contagion, there ought to prevail a considerable uniformity. We have long laid it down in our own minds, that the fevers resulting from marsh miasmata are not all of the same nature, but are considerably modified by the locality of the cause. The miasmata of Batavia for instance, will produce a disease differing in many points from the fevers of Walcheren or Philadelphia; and it has been proved, that acclimation to the miasmata of one place is no protection from those of another. Mr. Johnson has stated that veterans in India, and even the blacks, fell under the malignant influence of the miasmata of Batavia, though they were, in a great measure, proof against those of Bengal, Bombay, and other sickly parts of the East. Indeed, it is quite reasonable to suppose that febrific miasmata must differ essentially in different situations, and that the fevers they produce must vary with their causes.

The epidemic of Cadiz in 1810, at which time Sir James was head of the medical department there, is very cursorily passed over; in fact, no account at all is given of it, except that it was similar to the fever of 1800, which Sir James did *not* see! We must say, that Sir James has evinced rather too much modesty, in keeping back the whole of his own *personal* observations, while he has been so very liberal in his quotations from our good friends the Dons.

*Epidemic of* 1813—In the month of August, 1813, the heat varied at Cadiz from 81° to 91° Fahrenheit, and sometimes rose to 95°, with an easterly wind and cloudy atmosphere. This temperature was considerably above that of the same period in other years. Early in the month of September, a fever of a suspicious nature made its appearance in the well known abode of filth and poverty, *the Barrio of St. Maria,* and soon afterwards spread to other quarters of the town. A report of our author's on this occasion induced the British troops to evacuate Cadiz, and encamp eight miles distant, while the government taking alarm, decamped also. It is remarkable, that on this occasion, a committee of the principal medical men of the City, excepting three (Aregula, Flores, and Gonzales) gave in a certificate that no pestilential disorder existed at the time.

The cases, however, which our author witnessed during this epidemic, appear to bear a most striking resemblance to those of *Batavia,* related in Mr. Johnson's work on Tropical Climates, both as to symptoms and mortality.

We shall here introduce a few specimens of the Spanish *practice* in these fevers. The *general* treatment was on the plan of Aregula; but the singular success which is said to have attended the practice of La Fuente deserves consideration. His plan was to force the patient, if possible, to swallow six or eight ounces of bark within the first forty-eight hours of the disease. At the village of Los Barrios, a few miles distant from St. Roque, ninety patients took the bark within the first *eight hours* of the fever, of whom *none* died, excepting one man, carried off by a gouty affection. Of eight patients, to whom it was administered between the eighth and tenth hour, all recovered. Of five, who began between the twelfth and twenty-fourth hour, three recovered and two died. Of twenty, who did not take it till the second day, thirteen recovered and seven died. Of seventeen, who waited till the third or fourth day, eight recovered and nine died. And lastly, out of eighty-nine persons who made no use of the bark, but took other remedies, only twenty-two recovered and seventy-seven died.

So much were the judicious part of the inhabitants assured of these facts, that they contended, as it were, who should take most bark in the first forty-eight hours, and who should begin earliest. A patient generally sent for half a pound or more of the bark, the moment he felt the first shiver, and swallowed it in large spoonfuls to the amount of half an ounce or an ounce every two hours, without losing time, or allowing himself rest or sleep, either by night or by day. Many patients took ten, twenty, or even thirty ounces of it in a few days. Without being advocates for such a practice, we are strongly disposed to believe, that the effects of such a quantity of bark in the *very first moments of fever*, might really have been such as are represented; for we must recollect, that *then*, no great inequilibrium has taken place in the balance, either of the circulation or excitability; and that from the strong sympathy existing between the stomach and all other parts of the system, such an impression might have been made on the constitution, by the above extraordinary plan, as to arrest the further progress of the fever, in the same way as the cold affusion has checked the febrile catenation in limine. It is evident, however, that *after* any determination has taken place towards an organ; or in other words, *after* the balance of the circulation is much broken, the plan in question would be certain destruction. On this account also, it must rather be a self-adapted than a prescribed mode of treatment, since, in the latter case, the practitioner rarely sees disease in its very incipient movements.

"With respect to the treatment of the fever," says our author, "it may be remarked, that the general plan of cure consisted in early evacuating the stomach and bowels with the least possible irritation, and in supporting the force of the circulation." (We would rather say, preserving the due balance, or equal distribution of the circulation, than supporting the *force* of it in fever.) "It is to be observed, that an unusual torpor of the bowels attended this disease. Our practice in the military hospitals was principally directed to remove this, by means of six or ten grains of the subm. hydrarg. with or without an equal quantity of the ex. col. com. followed up by purging salts and injections. This plan generally produced copious alvine evacuations. The following day, a pill of two grains of the submuriate, with James's powder, was given every two hours. It was continued as long as there was an absence of untoward symptoms, or until the third day, when the tinct. cinchonæ was administered in doses of a tea spoonful, frequently repeated. The decoction, powder, extract, and tincture united, were afterwards given, according to the state of the patient's stomach: light and nutritious diet, in small quantities, were given during this time, with barley water or tea. Porter was equally grateful and beneficial.—In most cases, where the calomel was given, either with or without antimonial powder, it produced a lax state of the bowels, and a moisture on the skin, with immediate relief of the head-ache; and frequently with a diminution of the other febrile symptoms. I am persuaded, that, with proper management, it is, of all others, the most powerful and useful remedy in fevers, whether arising from human contagion or marsh miasmata." p. 406.

Bleeding did not succeed in this fever, according to our author, and emetics were improper. In conclusion, Sir James remarks—

"From all the observations that I had an opportunity of making in this disorder, and from which any practical inferences could be drawn, it appeared to me, that on the application of the poison to the stomach, which I apprehend to be the organ most directly affected, a morbid change took place in the gastric and other juices, which, by their peculiar stimulus on the nerves of the stomach and bowels, occasioned many of the febrile phænomena, and that the *bile* was the fluid principally acted upon. A consequent derangement in the ordinary secretion of the liver generally succeeded to a considerable extent, and thus the irritability of the system being increased, tended to keep up the fever. That such an effect is produced on the bile by poisons, human contagion, or marsh miasmata, will, I think, be admitted; and I have shewn, that in the treatment of the pestilential fever of Spain, and the bilious remittent of Holland, this predominance was strongly marked, and a correspondent principle of cure was adopted and constantly kept in view." p. 409.

Whatever may be thought of this reasoning, we believe the facts are correctly stated in the above passage. We are of opinion, however, that venæsection either had not a fair trial, or has not been fairly stated by those who have *yet* written on the fever in question, among the contagion party. The evidence of the utility of mercury is here unquestionable; and its liberal use, no doubt, made amends for the dread of the lancet.

We have now collected into a focus the most prominent traits and interesting facts which our author's work presents us, relative to those scourges which desolated the vales of Andalusia. We trust that we have thus formed for our numerous readers, who *cannot* peruse the original, an interesting epitome, that may prove no mean substitute for the volume before us; while to those who can afford the leisure and expence, we recommend the work as by no means undeserving a place in the philosophical wing of their library. To those who may be doomed to visit the peninsular shores of the Mediterranean, the publication of Sir James Fellowes is strongly recommended, as a very respectable companion to the volumes of Bancroft, Burnett, Irvine, and Pym. The work is written in a very gentlemanlike manner. Sir James will scarcely "hint dislike or hesitate a fault," even to the most prominent of his adversaries. Dr. Bancroft is the only man at whom he levels any thing like censure; and we suppose that able writer *must* come once more on the theatre, otherwise Messrs. Pym and Fellowes will consider him as fairly beaten from the field.

We have yet to notice that portion of the volume which is occupied with observations on the fever which afflicted our troops long after their return from the unfortunate expedition to the Scheldt. Sir James was one of the Physicians appointed to the Military Hospital at Colchester, and had a considerable field for observation. Most of his remarks, however, have been anticipated by other writers, who did not keep their portfolios so long in the back ground.

The frequency of *relapses* in this complaint, after the men had been far removed from the range of those causes originally producing it, excited much surprise among our practitioners, till *dissections* exhibited the most unequivocal proofs that visceral obstructions, particularly of the liver and spleen, were the lurking enemies which occasioned all the mischief. It was somewhat remarkable, that in these subsequent attacks, many were suddenly seized with head-ache and giddiness, commonly throwing themselves down on the bed, and becoming *comatose* in a few hours, without any increased action of the vascular system, or any external appearance to account for this symptom. In some of these patients, death took place in twenty-four or forty-eight hours from the time they first complained. Strangury, and excruciating pain across the loins, were also complained of in many instances. Post mortem examinations discovered enlargement and disease of the spleen and liver. In one case, the investing membrane of the spleen was converted into cartilage; and it was no uncommon occurrence to meet with it studded over with cartilaginous spots. The largest spleens, Sir James observed, were about four pounds in weight. The liver was almost universally diseased; the gall-bladder containing, in most instances, black and thick bile. No extraordinary morbid change was remarked in the brain of those patients who remained in a state of stupor previous to death, or who died in the cold fit of the intermittent.

The dissections led to a wise and successful mode of treatment, which hinged on the idea of congestion, or other organic derangement in the viscera above mentioned.

In one instance, Sir James remarked the glassy eye peculiar to the *Bulam* fever.

"The general plan of treatment, at first, was to open the body by means of five or six grains of the submuriate of mercury, with an equal quantity of the ex. col. comp. followed up by the infus. sennæ and sulph. magnes.; after which the submuriate and liq. ant. tart. were given every three or four hours in small doses, and continued, either with or without mercurial frictions on the side, according to the state of the patient and the period of the disorder, or until the mouth became *slightly* affected. Warm fomentations and blisters to the side, together with an anodyne at night, were also employed. In the *most obstinate cases*, it was found necessary to push the mercurial friction to some length, so as to induce ptyalism; and when there was appearance of remission, the *bark* was given, and *not till then*, in the form of decoction, with an aromatic: wine was also allowed in small quantities, according to the strength of the patient." p. 362.

As the cold weather advanced, the lancet was had recourse to with advantage. Cupping the regions of the liver and spleen was also found of great service.

A great proportion of the dropsies that were met with at Colchester evidently depended on affections of the liver and spleen. In these cases, a combination of mercury and squills, and in the convalescent stage, the bitter infusions with sulphate of iron, joined to the blue pill, formed the most successful treatment. Mercury was most beneficial when introduced slowly into the system, in the form of submuriate or pil. hydr.; and as soon as the mouth was affected, powdered squills were given three times a day, increasing the dose to seven or eight grains. The discharge of water which followed was astonishing, and then the mercurial was only given occasionally. The squills were exhibited as long as any water remained; and after each dose, the bitter infusion was administered with a scruple of the acetate of potash.

In this fever, says Sir James,

"Mischief has often arisen from the evacuating plan, particularly purgatives, not having been sufficiently followed up in the feverish complaints of soldiers, from a *mistaken notion of prevailing debility*; and the evil has been increased by the too early and liberal use of bark and wine."

It is not in the feverish complaints of soldiers only that mischief is done in this way. We every day see stimulants or placebos prescribed by *practical men* too, where the head is loaded, and the abdomen so tumid as scarcely to bear the slightest pressure! Yes; much as we hear of bleeding, purging, &c. in fevers and febrile complaints, *a majority*, we fear, of the *most active* part of the profession still trudge on in the usual routine which they pursued twenty or thirty years ago. We hope, however, even before our short span of existence is no more, to see a rational and vigorous practice in this extensive tribe of human ailments, instead of that temporising neutrality, or *delirious* system of stimulation, which at this moment is hurrying thousands to an untimely grave! The pages of this Journal will not cease to point out these evils till the remedy is applied; and we trust that the rising generation will feel the benefit and acknowledge it too.

Sir James's and his colleague Dr. Roberts's attention was drawn to the subject of purging, from an incident worth relating.

"The patient was a soldier, to whom the usual purgatives had been administered, which brought away a bilious discharge in some quantity; natural evacuations followed, and still the irritation of fever was kept up without any manifest cause. After an interval of two or three days, the calomel and purging mixture were again repeated, and produced a copious discharge of *a black colour, and viscid.* Immediately after this dark-coloured matter passed off, the patient felt relieved, and the feverish symptoms yielded readily. From the state in which we so often found the gall-bladders of the men who died of the prevailing complaint *full of black bile, of the consistence and colour of pitch, sometimes as viscous as bird-lime, and at others as fluid as treacle,* and in several of whom there had been evident inflammation of the liver and spleen, we concluded that this morbid bile had contributed to keep up the irritation of the system, and that it was a principal cause of the phænomena which were observed in this fever." p. 980.

These gentlemen we believe concluded justly; and most earnestly do we exhort our brethren in the profession to bear the above passage in their minds when they approach the bed-side of fever! No small number of our delicate practitioners are too polite to examine the contents of a close-stool, and are more intent on the less disagreeable task of serving the female by-standers with a sufficient quantum of *small-talk,* in order to enhance their consequence among the gossipping groups of society afterwards. These are not the men, however, who will ever contribute to the reputation of science, or the mitigation of human misery! Their fungous fame will expire even before themselves; while the honest exertions of genius and industry will be transmitted to posterity as the purest and most grateful incense to their departed spirits!

We now take leave of our author, whose work we have *attentively perused and faithfully delineated*; a duty which we have ever imposed on ourselves in the arduous task of criticism, and which shall always be the paramount rule of our conduct. In the publication before us, we have seen little to censure, something to admire, and much to interest our feelings. If our readers can conscientiously say as much of our critique, our labour is more than repaid.

Since finishing the above critique, we learn that the question respecting the "*Bulam Fever*" has become a national one. Mr. Pym's work, which we lately reviewed, has been sent by Government to the various Naval Medical Practitioners, who, from local experience, can give information on the points under discussion, and they are ordered to report thereon. We much doubt whether this mode of proceeding is that which is best calculated to elicit a fair and true opinion from those officers. Both sides of the question should undoubtedly be submitted to a jury before a verdict is given. The simple, unadorned account of the Gibraltar fever, inserted in our Journal for August last, from the pen of Mr. Amiel, who witnessed the whole of the epidemics at this fortress, and whose statements are in opposition to Mr. Pym, should be carefully perused before the sophistry and casuistry of party writers are suffered to exert their influence on the mind.

We trust that Government will be slow in deciding on this important question, till *the whole truth, and nothing but the truth*, can be sifted out of the mass of heterogeneous evidence which, on this occasion, will flow from various quarters. Those who are interested in this question, from local knowledge of the fever or fevers alluded to, are earnestly requested to transmit to the Editors of this Journal such information as they possess, which shall appear under their own names, or anonymous, to the public, as may be desired.

EDITORS.

