# Corrigenda

**Published:** 1816-02

**Authors:** 


					21, Motto, for tryriusve, read lyriusve
29, 1. 4, from bottom, after exist infert not

				

